# Oncology Provider and Patient Perspectives on a Cardiovascular Health Assessment Tool Used During Posttreatment Survivorship Care in Community Oncology (Results from WF-1804CD): Mixed Methods Observational Study

**DOI:** 10.2196/65152

**Published:** 2025-03-06

**Authors:** Chandylen L Nightingale, Emily V Dressler, Maura Kepper, Heidi D Klepin, Simon Craddock Lee, Sydney Smith, Aylin Aguilar, Kimberly D Wiseman, Stephanie J Sohl, Brian J Wells, Joseph A DeMari, Alyssa Throckmorton, Lindsey W Kulbacki, Jenny Hanna, Randi E Foraker, Kathryn E Weaver

**Affiliations:** 1 Department of Social Sciences and Health Policy Wake Forest University School of Medicine Winston-Salem, NC United States; 2 Department of Biostatistics and Data Science Wake Forest University School of Medicine Winston-Salem, NC United States; 3 The Prevention Research Center Washington University in St. Louis St. Louis, MO United States; 4 Department of Internal Medicine Wake Forest University School of Medicine Winston-Salem, NC United States; 5 Department of Population Health School of Medicine University of Kansas Kansas City, KS United States; 6 Qualitative and Patient-Reported Outcomes Shared Resource Wake Forest University School of Medicine Winston-Salem, NC United States; 7 Section on Gynecologic Oncology Wake Forest University School of Medicine Winston-Salem, NC United States; 8 Baptist Memorial Health Care/Mid-South Minority Underserved NCORP Memphis, TN United States; 9 ThedaCare Regional Cancer Center Appleton, WI United States; 10 Mercy Hospital Fort Smith Fort Smith, AR United States; 11 General Medical Sciences Institute for Informatics Washington University in St. Louis St. Louis, MO United States

**Keywords:** cancer, cardiovascular health, cancer survivors, community oncology, electronic health record integration, provider acceptability, patient-provider, assessment tool, electronic health records, clinical decision support, surveys, interviews, survivors, Automated Heart-Health Assessment

## Abstract

**Background:**

Most survivors of cancer have multiple cardiovascular risk factors, increasing their risk of poor cardiovascular and cancer outcomes. The Automated Heart-Health Assessment (AH-HA) tool is a novel electronic health record clinical decision support tool based on the American Heart Association’s Life’s Simple 7 cardiovascular health metrics to promote cardiovascular health assessment and discussion in outpatient oncology. Before proceeding to future implementation trials, it is critical to establish the acceptability of the tool among providers and survivors.

**Objective:**

This study aims to assess provider and survivor acceptability of the AH-HA tool and provider training at practices randomized to the AH-HA tool arm within WF-1804CD.

**Methods:**

Providers (physicians, nurse practitioners, and physician assistants) completed a survey to assess the acceptability of the AH-HA training, immediately following training. Providers also completed surveys to assess AH-HA tool acceptability and potential sustainability. Tool acceptability was assessed after 30 patients were enrolled at the practice with both a survey developed for the study as well as with domains from the Unified Theory of Acceptance and Use of Technology survey (performance expectancy, effort expectancy, attitude toward using technology, and facilitating conditions). Semistructured interviews at the end of the study captured additional provider perceptions of the AH-HA tool. Posttreatment survivors (breast, prostate, colorectal, endometrial, and lymphomas) completed a survey to assess the acceptability of the AH-HA tool immediately after the designated study appointment.

**Results:**

Providers (n=15) reported high overall acceptability of the AH-HA training (mean 5.8, SD 1.0) and tool (mean 5.5, SD 1.4); provider acceptability was also supported by the Unified Theory of Acceptance and Use of Technology scores (eg, effort expectancy: mean 5.6, SD 1.5). Qualitative data also supported provider acceptability of different aspects of the AH-HA tool (eg, “It helps focus the conversation and give the patient a visual of continuum of progress”). Providers were more favorable about using the AH-HA tool for posttreatment survivorship care. Enrolled survivors (n=245) were an average of 4.4 (SD 3.7) years posttreatment. Most survivors reported that they strongly agreed or agreed that they liked the AH-HA tool (n=231, 94.3%). A larger proportion of survivors with high health literacy strongly agreed or agreed that it was helpful to see their heart health score (n=161, 98.2%) compared to survivors with lower health literacy scores (n=68, 89.5%; *P*=.005).

**Conclusions:**

Quantitative surveys and qualitative interview data both demonstrate high acceptability of the AH-HA tool among both providers and survivors. Although most survivors found it helpful to see their heart health score, there may be room for improving communication with survivors who have lower health literacy.

**Trial Registration:**

ClinicalTrials.gov NCT03935282; http://clinicaltrials.gov/ct2/show/NCT03935282

**International Registered Report Identifier (IRRID):**

RR2-https://doi-org.wake.idm.oclc.org/10.1016/j.conctc.2021.100808

## Introduction

Survivors of many common early-stage cancers are now more likely to die of cardiovascular disease than cancer, elevating the importance of addressing cardiovascular health (CVH) in routine survivorship care [1-6]. Over 90% of survivors have multiple cardiovascular risk factors [7], increasing their risk of both poor cardiovascular and cancer outcomes [8-16]. Compared to the general population, survivors of cancer have poorer CVH [17,18]. Over 85% of survivors do not meet the American Heart Association’s healthy standards in multiple CVH components (BMI, physical activity, diet, smoking, blood pressure, cholesterol, and glucose) [7,19], many of which increase the risk for both cardiovascular disease and cancer [8,20]. Accordingly, better CVH among survivors is associated with improved survival [21] and reduced risk of both cardiovascular disease [20,22,23] and cancer recurrence [12-14].

Despite Institute of Medicine recommendations for prevention efforts and care coordination for survivors of cancer [24,25], up to 20% of survivors of breast and colorectal cancers may not see a primary care provider [26,27], heightening their risk for lack of preventive services and poor comorbidity management [27-29]. Claims data reveal that only 31%-39% of survivors of breast cancer received cholesterol screening, significantly fewer than women without breast cancer matched on age, ethnicity, sex, region, and comorbidity [29]. Together, these findings emphasize the importance of addressing CVH during routine oncology survivorship care. Both the American Society of Clinical Oncology [30] and the National Comprehensive Cancer Network guidelines [31] recommend cardiovascular risk assessment and discussion for patients with cancer. In our prior work [32] with 20 oncologists, 95% (n=19) reported CVH discussions to be “somewhat” or “very” important; however, 58% only “rarely” or “sometimes” discuss CVH with their patients [33]. Further, nearly 35% of survivors of cancer do not receive assistance from a health care provider for CVH-related lifestyle changes [2]. Similarly, fewer survivors who are at increased risk for health complications report provider discussions about CVH-related lifestyle behaviors (ie, physical activity, diet, and smoking) compared to those with no cancer history [34].

To address these gaps in posttreatment survivorship care and promote guideline adherence, our team developed and deployed a novel, easy-to-use, electronic health record (EHR)–embedded CVH assessment tool, the Automated Heart-Health Assessment (AH-HA) tool. This tool was first implemented in primary care and now incorporates EHR data on receipt of cancer treatments with cardiotoxic potential alongside a visual, interactive display of CVH risk factors, automatically populated from the EHR [35-37]. Before proceeding to future implementation trials, it is critical to establish the acceptability of the tool among oncology providers and survivors [38]. As part of a larger pragmatic trial to test and evaluate AH-HA in survivorship care [39], among practices randomized to the AH-HA tool, we assessed the acceptability of the AH-HA tool among both patients and providers during routine oncology care, along with provider perceptions of potential sustainability.

## Methods

### Ethical Considerations

This study (WF-1804CD) was approved by the National Cancer Institute (NCI) Central institutional review board (IRB). Each participating institution granted authority to the NCI Central IRB to serve as the IRB of record for NCI Community Oncology Research Program (NCORP) studies, in accordance with the National Institute of Health’s single IRB policy. All participants provided consent. NCORP is a national network of community oncology practices with infrastructure to support the recruitment of patients to clinical trials [40]. This study was facilitated through the Wake Forest NCORP Research Base (UG1CA189824). Study data were de-identified. Providers were offered a $10 gift card upon completion of the posttraining survey and a $20 gift card for participating in the qualitative interview. Survivors received a $10 gift card upon completion of the acceptability survey.

### Study Eligibility and Recruitment Procedures

Weaver et al [39] show the complete eligibility criteria and methods for the larger randomized trial. NCORP practice eligibility criteria included (1) use of the Epic EHR, (2) willingness to incorporate the AH-HA tool in their EHR, (3) having two or more providers willing to be trained and use AH-HA, and (4) identified combined providers saw 100 or more potentially eligible patients for follow-up in prior 6 months. Providers were recruited and consented by cancer care delivery research leads within their practice. Eligible providers included physicians and advanced practice providers (nurse practitioners and physician assistants) willing to complete the AH-HA provider training. This manuscript focuses on providers within practices randomized to use the AH-HA tool in the pragmatic trial [39]. To identify eligible survivors, staff at NCORP sites screened clinic schedules and reviewed survivors’ medical records. Survivors were contacted by phone, patient portal, or in-person and were eligible if they were at least 6 months post potentially curative cancer treatment for breast, prostate, colorectal, or endometrial cancers or Hodgkin and non-Hodgkin lymphomas and scheduled for a routine cancer-related follow-up care visit.

### AH-HA Training and Intervention

A full description of the AH-HA tool and provider training is available in the protocol paper [41]. In brief, providers completed two 30-minute video trainings prior to the practice enrolling patients. The training covered (1) the importance of addressing CVH as part of routine posttreatment follow-up care for survivors of cancer, (2) the basics of the American Heart Association’s Life’s Simple 7 CVH factors [9] and overall CVH metric, (3) navigation of the AH-HA tool within the EHR, and (4) how to use the tool to guide discussions with survivors. The AH-HA tool was launched using a best practice alert for enrolled patients during a routine posttreatment outpatient oncology visit. Providers could choose to use the tool or not in accordance with their clinical judgment; examples of reasons for nonuse may include a competing clinical demand (eg, new symptom or concern for recurrence), patient distress, or perception that the patient would not be receptive to or benefit from a discussion (eg, in the unlikely case that all factors were ideal). Five of the CVH factors were automatically populated from the EHR when available (BMI, smoking, blood pressure, cholesterol, and hemoglobin A_1c_ or blood glucose); physical activity and diet data were collected on paper and entered directly into the tool by the provider. AH-HA color codes each CVH factor as red (poor), yellow (intermediate), or green (ideal) according to Life’s Simple 7 classification framework [9] and also provides a total CVH score. Interactive slider bars can be used to demonstrate how improvements in CVH factors can lead to shifts in the categorization and overall CVH score. A second tab included information about the patient’s receipt of cancer treatments with cardiotoxic potential (ie, anthracyclines, antimetabolites, hormone therapy, aromatase inhibitors, monoclonal antibodies, antimicrotubule agents, alkylating agents, and radiation) [3,42,43].

### Data Collection and Measures

Providers and survivors provided information about sex, age, race, and ethnicity. Survivor cancer type and time since diagnosis were abstracted from the EHR. Survivor’s health literacy was also assessed with 1 item (“How confident are you filling out medical forms by yourself?”) with response options ranging from not at all confident to extremely confident. Prior research has demonstrated that this 1 item is effective at identifying health literacy skills [44]. Provider items also included provider type (physician, nurse practitioner, and physician assistant), years in current position, time spent providing direct patient care, time spent using the EHR for direct individual patient care, and proficiency with current EHR. Providers completed 2 surveys: one immediately after participating in the initial AH-HA training (posttraining survey) before participant enrollment and one after 30 patients were enrolled at the practice (postenrollment survey). Provider surveys assessed the acceptability of the training and AH-HA tool, and preferences for when and how often to use the AH-HA tool in the cancer treatment trajectory. Survivors completed one survey to assess the acceptability of the AH-HA tool immediately after the designated routine oncology appointment. Data collection occurred from December 2020 to March 2023. Survey items developed by our team are available upon reasonable request.

#### Provider Perspectives on Training

We developed a 7-item survey for the purpose of this study to assess various aspects of acceptability (eg, “The AH-HA training provided useful information about the importance of addressing CVH with cancer survivors” and “The AH-HA provider training will help me be more effective when discussing cardiovascular health with survivors”) with response options ranging from 1=strongly disagree to 7=strongly agree. A composite score was calculated using the average of all 7 items. Also, 1 item assessed the acceptability of the AH-HA training duration (response options ranging from too short to too long). An additional item assessed comfort in discussing CVH with survivors following the training (“Please indicate your level of comfort discussing CVH with your posttreatment, good prognosis patients”) with a 5-point Likert-scale (not at all comfortable to very comfortable). In a separate follow-up survey, providers were asked 1 item retrospectively about their preparedness to use the AH-HA tool at the time they completed the training (“Following the provider training, how prepared were you to use the AH-HA tool with patients?”). Response options included not at all prepared, somewhat prepared, and very prepared.

#### Provider Perspectives on the AH-HA Tool

Six items, used in prior work [32,36], assessed aspects of provider acceptability of the AH-HA tool (eg, “The information AH-HA provides is useful” and “AH-HA helps me be more effective”) with response options ranging from 1=strongly disagree to 7=strongly agree. A composite score was calculated using the average of all 6 items. We further assessed acceptability using items from the Unified Theory of Acceptance and Use of Technology (UTAUT) survey [45]. Specifically, 15 items assessed the performance expectancy (eg, “The AH-HA tool is useful in my job”), Effort expectancy (eg, “I find the AH-HA tool easy to use”), attitude toward using technology (“Using the AH-HA tool is a good idea”), and facilitating conditions (eg, “I have the resources necessary to use the AH-HA tool”) domains of the UTAUT survey [5,45]. Response options ranged from 1=strongly disagree to 7=strongly agree. We calculated scores for each domain using the average of domain items [45]. An additional item that was developed for this study to assess potential sustainability asked providers to report the timing and frequency they would like to use the AH-HA tool (“After the study ends, how often would you like to use the AH-HA Tool when providing care to patients during: (1) initial treatment planning, (2) active treatment, and (3) posttreatment survivorship care”) with response options including never or almost never, seldom or about half the time, most of the time, and always or almost always.

#### Survivor Acceptability

Five items previously used in our pilot work assessed overall acceptability of the AH-HA tool (eg, “I liked the heart health tool I used today with my provider” and “It was helpful to see my heart health score”) with a 5-point Likert scale ranging from strongly disagree to strongly agree [32].

Providers also participated in a semistructured qualitative interview conducted via telephone at the end of patient enrollment at their practice to further understand perceptions of the AH-HA tool. Examples of interview content include the impact of AH-HA on the provider’s practice (“How do you think having access to the tool impacted your practice, if at all?”), patients’ responses to the tool (“How did patients respond or react to the tool?”), recommended changes to the tool (“What changes would you make to AH-HA so it will work effectively in your setting?”), impact on care provided to patients (“Overall, do you feel the tool helped improve the care you provide to patients? Why/why not?”), and benefits and drawbacks to continuing to use the tool (“What benefits and drawbacks do you see in continuing to use AH-HA in your practice after the study is complete?”). A full list of interview questions is available upon request.

Interviews were conducted by 2 trained qualitative research team members from the Qualitative and Patient-Reported Outcomes (Q-PRO) Shared Resource of the Atrium Health Wake Forest Baptism Comprehensive Cancer Center. Interviews lasted an average of 20 minutes and were audio recorded.

### Analyses

Descriptive statistics were quantified with mean (SD) and frequency (%) for continuous and categorical outcomes respectively. Figures display mean and corresponding 95% CIs for providers’ answers on a 1-7 scale. Total scores for scales are quantified with mean (SD) and range. Univariate associations of demographics characteristics (age, sex, race, ethnicity, and health literacy) and cancer type (breast, colorectal, prostate, endometrial, and lymphoma) with acceptance of the AH-HA tool (using the following items: (1) “It was helpful to see my heart health score” and (2) “I would like to use this tool to talk about my heart health with my oncology provider at a future appointment”) were tested using Fisher exact tests. *P* values less than .05 were considered statistically significant.

Qualitative interviews were analyzed in collaboration with the Q-PRO Shared Resource of the Atrium Health Wake Forest Baptism Comprehensive Cancer Center. The interview audio was transcribed verbatim and 2 Q-PRO teammates and coauthors (AA and KW) reviewed the transcripts and developed a draft codebook. The study team reviewed the codebook and provided input, which was incorporated into a new version of the codebook. Transcripts were imported into ATLAS.ti [46] and the codebook was tested by coding several transcripts and revised as necessary. All interviews were independently coded by 2 Q-PRO teammates and coauthors (AA and KW) and compared; any discrepancies were discussed and resolved. Once all transcripts were coded, code reports were run and summaries for each report were written. Summaries for provider’s perceptions of the AH-HA tool were synthesized and analyzed for patterns and themes.

## Results

### Overview

In total, 17 providers were recruited for the pragmatic trial to participate in the intervention arm; 1 provider did not use the AH-HA tool and 1 provider did not complete surveys. Thus, we report results on the 15 providers who used the AH-HA tool and completed the surveys from 4 community oncology practice groups (25% of practices located in the Midwest, 75% or practices located in the South; 25% minority or underserved NCORP, and 50% designated critical access hospital). A total of 13 providers (87%) completed the posttraining survey and 15 (100%) completed the postenrollment survey. Among the 15 providers who used the tool, together they saw 296 survivors (46% of survivors participating in the larger randomized trial). Of these, 245 reported seeing the AH-HA tool (33 did not see the tool and 18 were unknown).

### Provider and Survivor Characteristics

#### Overview

Providers included physicians (n=8, 53%; Table 1), nurse practitioners (n=6, 40%), and a physician assistant (n=1, 7%). Most providers (n=10, 67%) reported spending 76%-100% of their time providing direct patient care and more than half (n=8, 53%) reported spending 76%-100% of their time using the EHR for direct patient care. Most providers (n=11, 73%) reported that they were “very proficient” with their current EHR.

**Table 1:** Provider characteristics (N=15).

Characteristics			Values
**Sex, n (%)**			
	Female	11 (73)	
	Male	4 (27)	
**Age (years), n (%)**			
	26-35	2 (13)	
	36-45	6 (40)	
	46-55	3 (20)	
	65 and older	1 (7)	
	Unknown	3 (20)	
**Race, n (%)**			
	Asian	3 (20)	
	White or Caucasian	11 (73)	
	Not reported	1 (7)	
**Ethnicity, n (%)**			
	Non-Hispanic	13 (87)	
	Not reported	2 (13)	
**Provider role, n (%)**			
	Physician	8 (53)	
	Nurse practitioner	6 (40)	
	Physician assistant	1 (7)	
**Years in current position, n (%)**			
	1-5	5 (33)	
	6-10	5 (33)	
	11-20	4 (27)	
	More than 20	1 (7)	
**Time spent providing direct patient care (%), n (%)**			
	51-75	5 (33)	
	76-100	10 (67)	
**Time spent using EHR^a^ for direct individual patient care (%), n (%)**			
	26-50	3 (20)	
	51-75	4 (27)	
	76-100	8 (53)	
**Proficiency with current EHR, n (%)**			
	Very proficient	11 (73)	
	Somewhat proficient	3 (20)	
	Neutral	1 (7)	
Number of survivors that used AH-HA^b^ per provider, mean (SD); range			19.7 (17.1); 2-56

^a^EHR: electronic health record.

^b^AH-HA: Automated Heart-Health Assessment.

Survivors (N=245; Table 2) completed treatment for breast (n=230, 93.9%), endometrial (n=1, 0.4%), or colorectal (n=9, 3.7%) cancers, or lymphoma (n=5, 2%) and were mostly female (n=239, 97.5%). Most survivors were White or Caucasian (n=203, 82.9%) and 13.1% (n=32) were Black or African American. Overall, 5.3% were Hispanic or Latino (n=13). Survivors were an average age of 61 (SD 10.9) years and most commonly married or living as married (n=176, 71.8%). Most survivors had a college degree (n=111, 45.3%) or some college including vocational or technical school (n=82, 33.5%). The median time since diagnosis was 3.6 (IQR 2.1-5.2) years.

**Table 2:** Survivor characteristics (N=245).

Characteristics		Values
**Sex, n (%)**		
	Female	239 (97.5)
	Male	6 (2.5)
**Age (years), n (%)**		
	18-39	9 (3.7)
	40-64	131 (53.5)
	65-74	84 (34.3)
	75 and older	21 (8.6)
**Race, n (%)**		
	American Indian or Alaskan Native	1 (0.4)
	Asian	3 (1.2)
	Black or African American	32 (13.1)
	White, non-Hispanic or Latino	193 (78.8)
	White or other or unknown, Hispanic or Latino	13 (5.3)
	More than 1 race, not Hispanic or Latino	2 (0.8)
	Other or unknown, not Hispanic or Latino	1 (0.4)
**Ethnicity, n (%)**		
	Hispanic or Latino	13 (5.3)
	Not Hispanic or Latino	232 (94.7)
**Marital status, n (%)**		
	Married or living as married	176 (71.8)
	Single, divorced, separated, or widowed	69 (28.2)
**Education, n (%)**		
	High school or less	52 (21.2)
	Some colleges (including vocational or technical)	82 (33.5)
	College degree or more	111 (45.3)
**Cancer type, n (%)**		
	Breast	230 (93.9)
	Colorectal	9 (3.7)
	Endometrial	1 (0.4)
	Lymphoma	5 (2.0)
**Time since diagnosis (years)**		
	Median (IQR)	3.61 (2.14-5.22)
	Unknown (n=6)	N/A^a^

^a^N/A: not applicable.

#### Provider Perspectives on AH-HA Training

**Figure 1:**
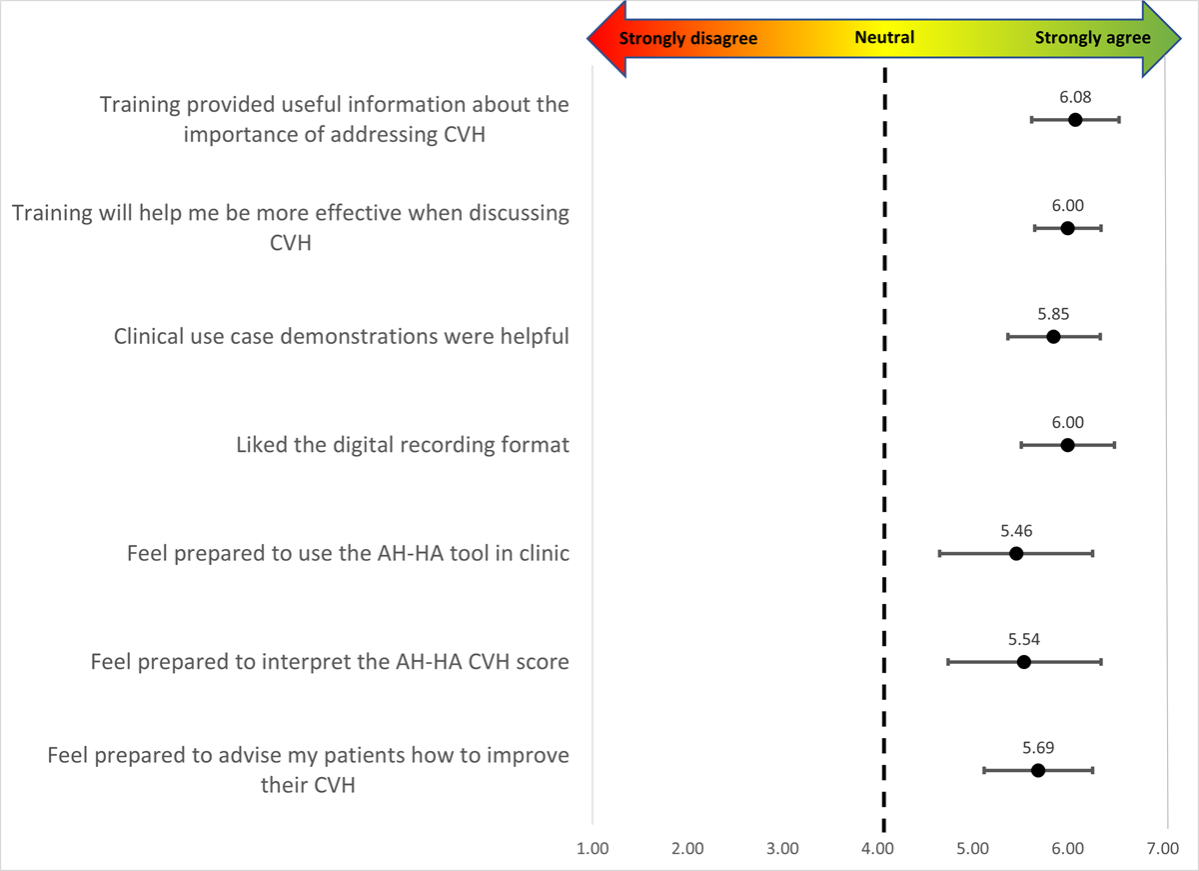
Provider acceptability of the AH-HA training session (N=13). AH-HA: Automated Heart-Health Assessment; CVH: cardiovascular health.

Figure 1 depicts provider training acceptability findings. Overall, providers reported high acceptability (mean 5.8, SD 1.0), with the highest item acceptability rating (mean 6.1, SD 0.8) for the following item: “The AH-HA training provided useful information about the importance of addressing CVH with cancer survivors.” Providers reported the lowest acceptability rating (mean 5.5, SD 1.3) for the following item: “I feel prepared to use the AH-HA tool in clinic with posttreatment, good prognosis patients.” More than half of providers reported that the duration of the AH-HA training was “about right” (n=7, 54%), followed by “a little too long” (n=4, 31%), “a little too short” (n=1, 8%), and “much too long” (n=1, 8%). At the conclusion of the training, all providers reported that they were somewhat (n=9, 69%) or very (n=4, 31%) comfortable discussing CVH with posttreatment patients with a good prognosis. When providers reflected on their preparedness after using the AH-HA tool, most reported they were “very prepared” (n=8, 57%) followed by “somewhat prepared” (n=6, 43%).

#### Provider Perspectives of the AH-HA Tool

**Figure 2:**
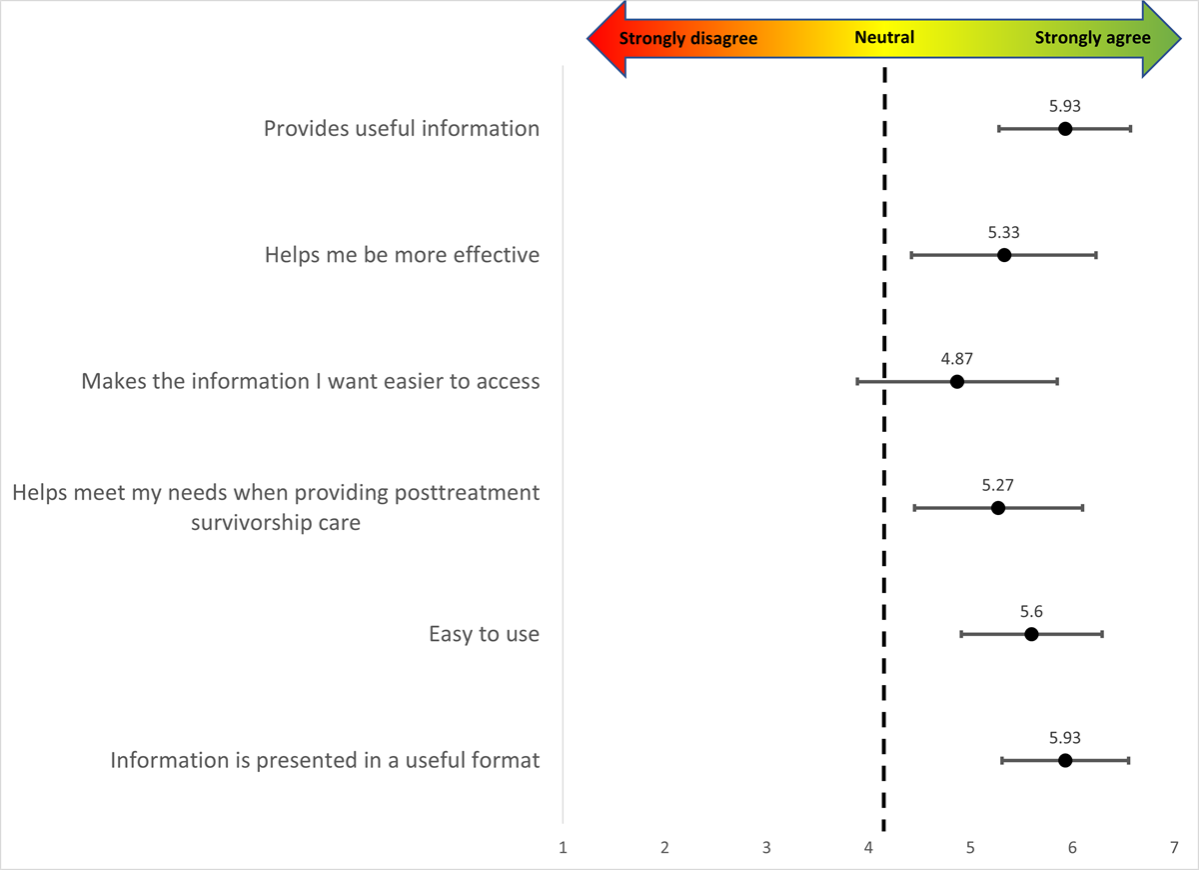
Provider acceptability of the AH-HA tool (N=15). AH-HA: Automated Heart-Health Assessment.

Quantitative surveys and qualitative interview data converged to demonstrate provider acceptability of the AH-HA tool. Figure 2 shows survey results; providers reported being satisfied with the AH-HA tool (mean 5.5, SD 1.4) with the highest rating for 2 items: “The information AH-HA provides is useful” (mean 5.9, SD 1.2) and “The information in AH-HA is presented in a useful format” (mean 5.9, SD 1.1); and the lowest rating (mean 4.9, SD 1.8) for the following item: “AH-HA makes the information I want easier to access.” Providers felt the interactivity and visuals provided in the AH-HA tool were particularly useful for patients. One provider stated, “I think the biggest thing is the visual aspect of the tool is really nice for them and the interactive-ness, the way you can slide the bars and show them if they achieve X, Y, or Z goal, how it can make a difference in their [CVH] score.” One provider mentioned that having these data available would allow them to easily track their patients’ progress: “it was something that, in a follow up visit, you could look—would be able to look back on to compare and talk with the patient and they can see how they made progress in this area or is there something we can continue to work on. It helps focus the conversation and give the patient a visual of continuum of progress.” Providers reported acceptability of AH-HA (Table 3) for the performance expectancy (mean 4.0, SD 2.0), effort expectancy (mean 5.6, SD 1.5), attitude toward using technology (mean 4.8, SD 2.1), and facilitating conditions (mean 5.5, SD 1.5) domains of the UTAUT. Related to performance, providers felt AH-HA helped them have deeper discussions of cardiovascular risk with patients.

**Table 3:** Provider acceptability for the performance, effort, attitude, and facilitating conditions domains of the UTAUT^a^ survey (N=13).

UTAUT domain	Mean (SD); range
Performance	3.98 (2.04); 1-7
Effort	5.62 (1.49); 2-7
Attitude	4.75 (2.06); 1-7
Facilitating conditions	5.54 (1.51); 2-7

^a^UTAUT: Unified Theory of Acceptance and Use of Technology.

One provider stated, “Before, our CVH approach might have been more of a blanket statement about you are a breast cancer survivor, and you may have increased cardiovascular risks, so you need to optimize your blood pressure, cholesterol with your primary care doctor and what not, but this is a much more thorough tool.”

Some providers noted already having these conversations with patients, which made them less receptive to the tool. For example, 1 provider stated, “because I already do that anyway, I think it was just kind of time-consuming…to actually do it and make extra time in the visit to go through that particular part on the computer and have them ask—or answer very specific questions when we really kind of discuss all of this anyway.” In contrast, favorable effort expectancy was supported qualitatively as some providers noted that the tool was “very simple to use” and “user friendly.” Providers did feel it could be easier to use if it required less “maneuvering” or having to go back and forth” within the EHR.

#### Potential Sustainability of the AH-HA Tool

When asked about using the tool after the study ended, most providers reported interest in using the AH-HA tool for posttreatment survivorship care (always or almost always: n=3, 21%; most of the time: n=7, 50%; seldom or about half the time: n=2, 14%; and never or almost never: n=2, 14%). There was less interest in using the tool for patients in active treatment or during initial treatment planning for which results were the same (most of the time: n=2, 14%; seldom or about half the time: n=5, 36%; or never or almost never: n=7, 50%).

#### Survivor Acceptability of the AH-HA Tool

**Figure 3:**
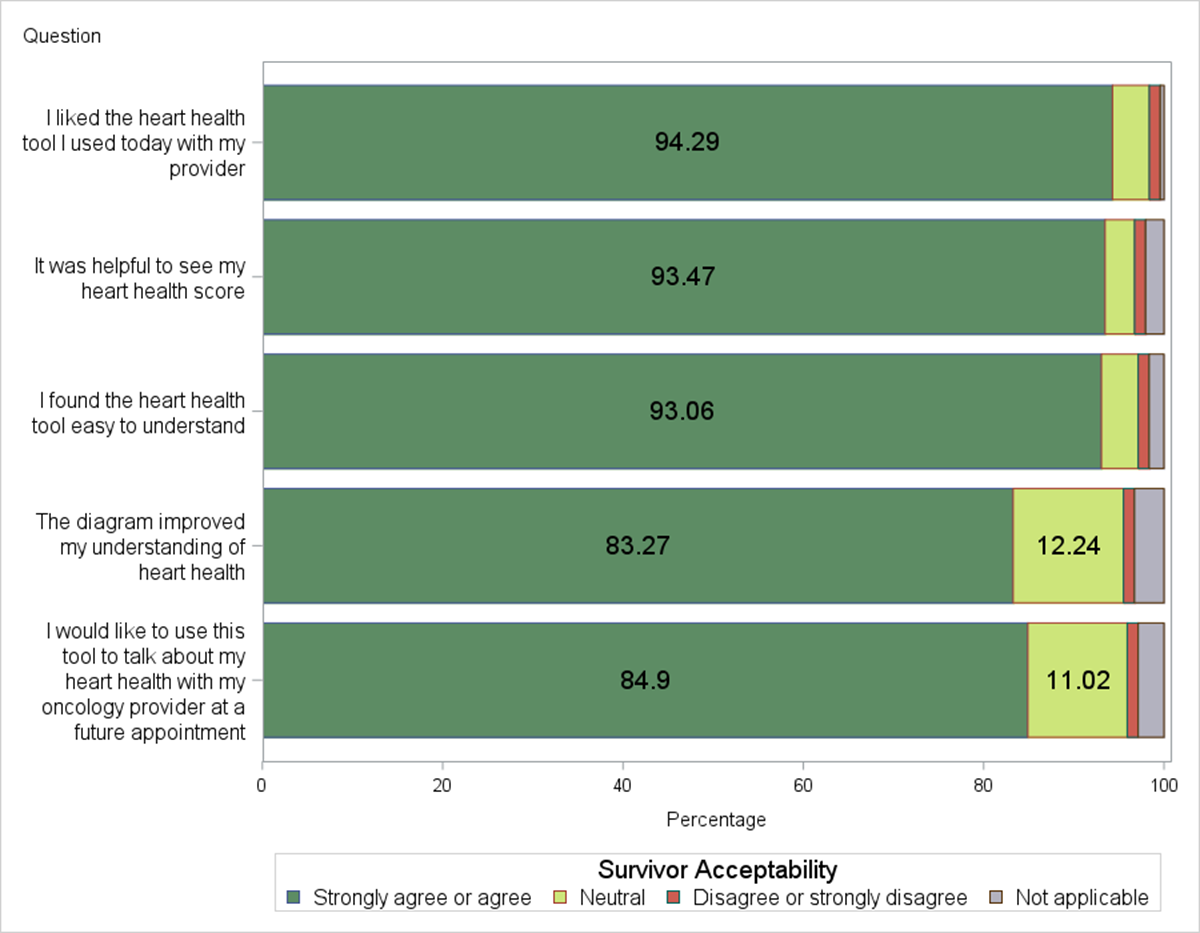
Survivor acceptability of the AH-HA Tool (N=15). AH-HA: Automated Heart-Health Assessment.

Figure 3 shows results for survivor acceptability of the AH-HA tool. Most survivors reported that they strongly agreed or agreed that they liked the AH-HA tool (n=231, 94.3%), it was helpful to see their heart health score (n=229, 93.5%), AH-HA was easy to understand (n=228, 93.1%), the picture or diagram (of CVH risk factors) improved their understanding of their heart health (n=204, 83.3%), and they want to use AH-HA to talk about heart health with their oncology provider at a future appointment (n=208, 84.9%).

#### Associations With Survivor Acceptability of the AH-HA Tool

Health literacy was the only survivor demographic characteristic significantly associated with survivors’ acceptability of the AH-HA tool. Survivors who indicated they were “extremely confident” filling out medical forms on their own (ie, high health literacy) strongly agreed or agreed that it was helpful to see their heart health score (n=161, 98.2%) compared to survivors with lower health literacy scores (n=68, 89.5%; *P*=.005). Yet, the perceived helpfulness of seeing the heart health score was generally high.

## Discussion

Our mixed methods results support the acceptability of the AH-HA CVH assessment tool when used as part of routine posttreatment oncology care in community settings. Survivors of cancer were positive about using the tool in the clinic with their provider. Both oncology physicians and advanced practice providers across 4 community practices reported favorable perceptions of the AH-HA training and use of the tool with survivors of cancer. This suggests AH-HA may be well received in a variety of survivorship care models [47] (eg, advanced practice provider-led survivorship clinics or follow-up with the treating physician).

The overall high acceptability among both patients and providers supports the further implementation of the AH-HA tool, with a continued focus on posttreatment survivors of cancer. Most providers reported they would prefer to use the AH-HA tool for posttreatment survivorship care, and that they would rarely use AH-HA for patients in active treatment or during initial treatment planning. While CVH is important at all points in the cancer treatment trajectory, providers may want to prioritize oncologic treatment during the treatment planning and active treatment phases, and transition to health promotion during the posttreatment survivorship phase. Providers may also perceive that patients are able to more effectively focus on health behavior change without the logistical and psychosocial challenges that are heightened during the treatment phase [48,49]. It is also possible that providers preferred to use the AH-HA tool due to the framing effect of the trial (eg, only posttreatment patients were eligible and the study was focused on survivorship).

Our prior usability assessments considered the tool’s appropriateness of the CVH tool for posttreatment survivorship care and we learned that oncology providers also wanted to see potential cardiotoxic treatments received by survivors [33], as incorporated in this study. Moving forward, if the tool is to be used for treatment planning, usability assessments should be repeated with consideration of possible future cardiotoxic effects of treatments incorporated in the design of the tool.

Despite overall positive feedback from both providers and survivors about the AH-HA tool, our results suggest there may be some room for improvement in communicating the heart health score to patients with lower health literacy. Provider training could be augmented to include tips for using the tool with patients who have different levels of health literacy along with scripts to help guide the discussion with patients. Research shows providers often overestimate patient’s literacy levels, and patients may be too embarrassed about their limited health literacy to ask questions [50-53]. Increasing the provider’s awareness of a survivor’s health literacy may be an important step prior to initiating the CVH discussion with survivors. Although we assessed health literacy in this study, this information was not shared with providers. One potential strategy is to include an assessment of survivor health literacy as part of the AH-HA tool to help inform the CVH provider-survivor discussion. Additionally, providing basic information prior to the appointment on the components of CVH and their impact on a patient’s overall health may better prepare patients for the upcoming discussion with the provider. Similarly, enhancing patient-facing information in the format of an after-visit summary of CVH recommendations may enhance understanding for patients with lower health literacy [54].

Other potential modifications to the tool may address provider desires for a more streamlined experience. Although most providers found the tool simple to use, others suggested refining AH-HA by requiring less maneuvering within the EHR, and quantitative findings suggested room for improving ease of access to desired information. One way in which the tool could be simplified for providers would be to collect the self-reported diet and physical activity data via the patient portal prior to the visit so that the data would be available in the EHR and callable by the tool. This method would be expected to streamline the use of the tool at the point of care if these data would not need to be manually entered into the tool. Such modifications may also impact providers’ perceptions of how AH-HA will impact their job performance and interest in using the AH-HA tool, which corresponds to the UTAUT domains for which providers reported the lowest means.

There were notable strengths to this study. Provider feedback on AH-HA acceptability included both quantitative and qualitative data to provide a fuller picture of both overall acceptability and specific characteristics of the AH-HA tool, consistent with reported strengths of mixed methods research [55]. In this study, these data were complementary and enhanced understanding of provider acceptability. This study was also strengthened by the assessment of perspectives from both providers and survivors as the “end users” from 4 community oncology practices, to inform the next steps, and promote sustainability for AH-HA when implemented widely [56,57]. One limitation of the present study is the predominant enrollment of breast cancer patients despite broad inclusion criteria. This likely reflects the specialization of enrolling providers and the patient mix with respect to cancer type within survivorship programs. Our study team has reported interest in CVH discussions among survivors of gynecologic cancers, yet we acknowledge a more diverse survivor sample is needed to determine the generalizability of these results [58]. Although 15 providers participated and used the AH-HA tool, 2 providers (13%) did not complete the posttraining survey for unknown reasons. Due to the overall high acceptability of AH-HA, there was limited variability in detecting potential differences in patient acceptability by sex, age, or race and ethnicity. Further, although the sample size for our provider key informants was sufficient for theme saturation as our analytic approach [59], it also limited us from making comparisons in acceptability by provider type.

Building upon our strong acceptability findings, the next step for this line of research is to test the AH-HA implementation package to promote guideline-concordant CVH assessment and discussion among a larger and more diverse sample of oncology providers and patients. Tailoring the CVH discussion to meet the needs of patients with higher and lower health literacy will be an important factor to consider in this future direction. It will also be important to assess the sustainability of the AH-HA tool in community practice.

## Abbreviations

AH-HA: Automated Heart-Health Assessment
CVH: cardiovascular health
EHR: electronic Health Record
IRB: institutional review board
NCI: National Cancer Institute
NCORP: National Cancer Institute Community Oncology Research Program
Q-PRO: Qualitative and Patient-Reported Outcomes
UTAUT: Unified Theory of Acceptance and Use of Technology
